# Health Benefit Package Revision Is an Art as Much as a Science – Lessons Learned on the Organization of the Appraisal Phase

**DOI:** 10.34172/ijhpm.8819

**Published:** 2025-03-11

**Authors:** Rob Baltussen, Mojtaba Nouhi, Andrew Mirelman, Sameen Siddiqi, Cassandra Nemzoff, Gavin Surgey, Stella Umuhoza, Saltanat Zhetibaeva, Baktygul Isaeva, Anna Vassal

**Affiliations:** ^1^Radboud University Medical Center, Nijmegen, The Netherlands.; ^2^Ministry of Health and Medical Education, Tehran, Iran.; ^3^World Health Organisation (WHO), Geneva, Switzerland.; ^4^Aga Khan University, Karachi, Pakistan.; ^5^London School of Hygiene & Tropical Medicine, London, UK.; ^6^University of Rwanda, Kigali, Rwanda.; ^7^Ministry of Health, Astana, Kyrgyzstan.

**Keywords:** Health Benefit Package, Appraisal Phase, Guidance

## Abstract

Many low- and middle-income countries are designing or revising their health benefit packages (HBPs), with appraisal—prioritizing services for reimbursement—being a critical phase. This occurs in a complex landscape of multiple criteria, multiple stakeholders, limited evidence, budget constraints, and tight timelines, varying across countries. Existing guidance documents do not fully address these complexities, requiring analysts to balance methodological rigor with practical constraints. This editorial highlights four key themes in organizing appraisal: decision-making structures, trade-offs between criteria, final recommendations, and the use of cost-effectiveness evidence, thresholds, and budgets. These emerged as central challenges in HBP revisions in Iran, Kyrgyzstan, Liberia, Pakistan, and Rwanda. We emphasize cross-country learning to address these challenges pragmatically, recognizing that high-quality, legitimate appraisal is as much an art as a science. More detailed documentation of appraisal processes is needed to refine HBP revision guidelines and strengthen priority-setting in health systems.

## Introduction

 Many low- and middle-income countries are either developing their first health benefit package (HBP) or revising existing packages to advance universal health coverage.^1,2^ An important phase in this process is appraisal, ie, the prioritization of services based on a combination of scientific judgments (evaluating evidence quality) and social considerations (interpreting evidence in the context of social values and their trade-offs). The appraisal phase typically involves a deliberative process and results in the development of recommendations for public funding of services to final decision-makers, such as Ministers of Health or publicly financed insurance funds.^3^

 Appraisal often takes place in a highly complex context, characterized by an interplay of different factors such as the need to evaluate multiple services simultaneously; to consider the voice and interest of numerous stakeholders including their quest for transparency to ensure the process is viewed as legitimate; to deal with limited local evidence on critical criteria like cost-effectiveness; to operate under an unclear cost-effectiveness threshold and/or budget constraint; all in a typically tight (political) timeline.^3^ Moreover, these factors manifest themselves differently across countries. For example, whereas Pakistan revised its entire HBP,^4^ Iran^5^ initially focused on multiple sclerosis control, involving stakeholders and assessing opportunity costs solely within this condition rather than the broader health sector.

 Existing guidance documents on HBP design and revision describe the appraisal phase at varying levels of detail but fail to account for the socio-economic-political realities involved.^1,6-8^ Policy-makers and (local or international) analysts involved in HBP revision processes (referred to as “project team” in the remainder of the text) require practical guidance on how to maintain methodological rigor in challenging contexts. We consider the development of HBP revision processes as an art as much as a science, as it involves creativity to tailor the methods to the mentioned challenges.

 This editorial describes four interrelated themes that warrant special attention in the organization of the appraisal phase, and are likely to be context-dependent, ie, the decision-making structure; trading-off decision criteria; making final recommendations; and using cost-effectiveness evidence, thresholds, and budgets. These themes emerged as key considerations in our work as national technical leaders or international advisors in organizing the appraisal phase during whole HBP revisions in Kyrgyzstan, Liberia,^9^ and Pakistan,^4^ as well as partial package revisions in Iran (focused on multiple sclerosis control)^5^ and Rwanda (focused on cancer control). We do not cover other phases of the overall decision-making process, such as the selection of services for evaluation, the choice of decision criteria and evidence synthesis, assuming these elements are already established. We acknowledge that while this paper focuses on HBP revisions, the principles apply to initial HBP development as well.

 The editorial includes notions on what can ultimately become practical guidance, based on our involvement in the countries mentioned, a recent review,^10^ interactions with multiple colleagues, and personal judgments. These notions are not exhaustive, as other countries may follow different processes. Over time, the combined experience will hopefully enhance the understanding of how appraisals are conducted in HBP revisions.

## Decision-Making Structure

 Countries may employ a single (national) advisory committee (AC) during the appraisal phase to develop recommendations for HBP revisions to the final decision-maker. However, this structure presents two key challenges. First, such committees often comprise generalists who require significant time to understand the complexities of different diseases. Second, while extensive stakeholder involvement in HBP revision can enhance the quality and legitimacy of decisions, it also makes the process more time- and resource-intensive.^3^

 To address these challenges, the countries we have worked with have adopted a two-staged appraisal process involving technical working groups (TWGs) organised by disease-clusters, in addition to the AC (whereby we acknowledge variation in terminology across countries)^3-5,9,10^ (Table).

**Table:** The Organisation of the Appraisal Phase in the Selected Countries

**Aspect**	**Kyrgyzstan**	**Liberia**	**Pakistan**	**Iran**	**Rwanda**
Scope of appraisal	Across all conditions.	Across all conditions.	Across all conditions.	Cluster by cluster (HBP revision on multiple sclerosis is finalized).	Cluster by cluster (HBP revision on cancers is finalized).
Decision-making structure	Two-staged process with TWGs and AC.	Two-staged process with TWGs and AC.	Two-staged process with TWGs and AC.	Two-staged process with TWGs and AC.	Two-staged process with TWGs and AC.
Trading-off decision criteria	Qualitative approach with cost-effectiveness prominently displayed on evidence summary sheet. TWGs interpreted evidence summary sheets and classified services into high, medium, and low priority categories without applying a budget constraint.	Qualitative approach with cost-effectiveness prominently displayed on evidence summary sheet. TWGs interpreted evidence summary sheets and classified services into high, medium, and low priority categories without applying a budget constraint.	Qualitative approach with cost-effectiveness prominently displayed on evidence summary sheet. TWGs interpreted evidence summary sheets and classified services into high, medium, and low priority categories without applying a budget constraint.	Qualitative approach with three pillars of criteria: quality of care, necessity and sustainability. The TWG interpreted evidence summary sheets and made budget-neutral recommendations on (conditional) reimbursement of selected services.	Qualitative approach with cost-effectiveness prominently displayed on evidence summary sheet. The TWG interpreted evidence summary sheets and prioritized basic, core and enhanced cancer services specifying coverage levels, initially without a budget constraint and subsequently with one.
Making final recommendations	Due to budget constraints, not all high-priority services could be included. To align with the available budget, the AC selected a subset of high-priority services for inclusion in the benefit package, following deliberations.	Due to budget constraints, not all high-priority services could be included. To align with the available budget, an additional prioritization was done based on cost-effectiveness. The AC made final recommendations following this ranking and deliberations.	Due to budget constraints, not all high-priority services could be included. To align with the available budget, the AC selected a subset of high-priority services for inclusion in the benefit package, specifying coverage and co-payment levels, and distinguishing between immediate and deferred implementation.	The TWG's recommendations were adopted following deliberations in the AC.	The TWG's recommendations were adopted following deliberations in the AC.
Use of cost-effectiveness evidence, thresholds, and budgets	Cost-effectiveness thresholds were used to classify the performance of services, while the budget constraint guided their inclusion or exclusion.	Cost-effectiveness thresholds were used to classify the performance of services, while the budget constraint guided their inclusion or exclusion.	Cost-effectiveness thresholds were used to classify the performance of services, while the budget constraint guided their inclusion or exclusion.	Cost-effectiveness thresholds were used to classify the performance of services, while the budget constraint guided their inclusion or exclusion.	Cost-effectiveness thresholds were used to classify the performance of services, while the budget constraint guided their inclusion or exclusion.

Abbreviations: AC, advisory committee; TWG, technical working group; HBP, health benefit package.

 TWGs typically included stakeholders such as health professionals, scientists, payers, and patient representatives with technical expertise and/or experience in the cluster under evaluation (eg, cancer treatment or cardiovascular disease management), with a mandate to trade-off decision criteria, classify services into priority categories and develop preliminary recommendations to the AC. The involvement of TWGs allows extensive stakeholder involvement and secures relevant experience into the appraisal phase, though it does cost more time and resources.

 The AC subsequently consolidates these recommendations into a unified set of final recommendations for the final decision-maker, explicitly considering the three dimensions of the universal health coverage cube: service inclusion/exclusion, population coverage, and co-payment levels, all within budget constraints. The composition of the AC varied tremendously between countries, and typically included stakeholders representing broad societal interests, such as representatives from ministries, health insurance schemes, private sector, public health specialists, ethicists, economists, and general patient representatives. To date, social participation has been absent from both the TWGs and the AC.

 Below, for sake of argumentation, we refer to this process. We thereby acknowledge nuances to this model, eg, that the AC may also trade-off decision criteria in developing their final recommendations but do not report on it here. As an alternative to the above governance structure in terms of AC and TWGs, countries can also decide to establish a single AC and consult disease-specific experts in their meetings. This approach would expedite the process, but it may compromise expertise and legitimacy.

## Trading-Off Decision Criteria

 TWGs can use several alternative approaches to interpret the evidence for a particular service on various decision criteria.

**Figure 1:**
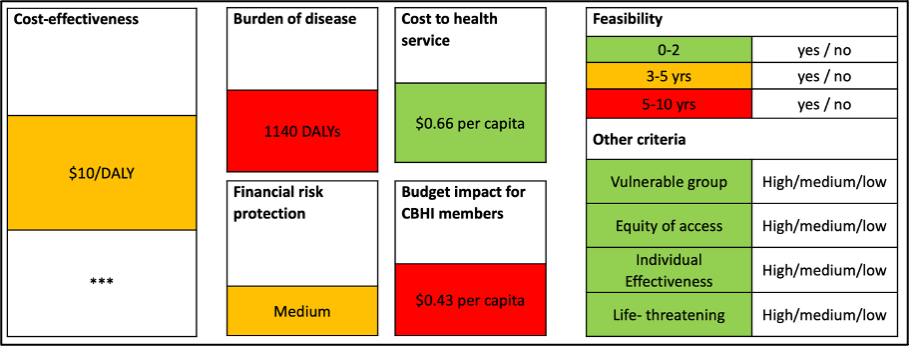
Evidence Summary Sheet, Hypothetical Example.^a,b^ Abbreviations: DALY, Disability-adjusted life year; CBHI, community-based health insurance. ^a^The performance of services on the various criteria is indicated by color-coding in which green coding reflects a favorable performance, amber a neutral performance and red an unfavorable performance. ^b^Uncertainty around cost-effectiveness is indicated by stars: * = large uncertainty, ** = medium uncertainty, *** = little uncertainty.

 In a *qualitative* approach, TWG members deliberate in an unstructured manner using an evidence summary sheet, which summarizes a service’s performance across all criteria (Figure 1).^11^ This approach is commonly used by several health technology assessment (HTA) agencies worldwide for evaluating single services and is often seen as convenient. The use of an evidence summary sheet also stimulates that all decision criteria are (equally) considered. The approach also carries risks, including stakeholder dominance, where stronger voices overshadow others, and increased cognitive burden when evaluating multiple services, potentially causing decision fatigue and weaker outcomes.^11^

 In a *quantitative* approach, traditionally labelled as multi-criteria decision analysis, TWG members provide scores (0-100) to the performance of service on each criterion, weigh each criterion, and analysts subsequently multiply scores by weights to estimate ranking results, followed by deliberation. The approach has much intuitive appeal, but conceptual requirements are often violated in practice, ie, (*i*) costs and/or cost-effectiveness are frequently included as a criterion whereas the analysis should only include criteria that reflect the societal value of a health service, not their resource use; (*ii*) decision criteria are often assumed to be compensatory, meaning strengths in one area can offset weaknesses in another. However, some health services, especially those failing to improve population health, cannot be justified by strengths in other areas; and (*iii*) deliberation is often inadequate which can lead to the neglect of potentially relevant non-quantifiable considerations.^11^ Yet, the approach may be instrumental when carried out correctly (detailed guidance is available elsewhere^12,13^) and is especially useful when many services are to be evaluated at the same time to reduce cognitive overload.

**Figure 2:**
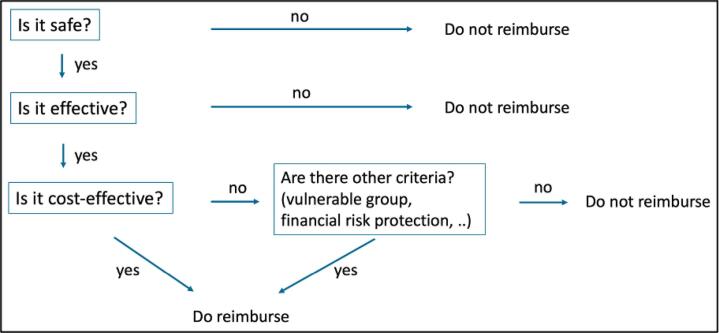
Decision Tree Approach.

 In a *decision-tree approach*, TWG members follow a hierarchical set of questions based on decision criteria,^11^ and is used by HTA agencies in the United Kingdom,^14^ the Netherlands,^15^ and Thailand.^16^ In this approach, cost-effectiveness typically serves as the central criterion, reflecting the health system’s primary objective of maximizing health outcomes, and other criteria are interpreted in relation to cost-effectiveness (such as whether the service targets a vulnerable population or provides protection against financial risks). Figure 2 illustrates the approach in a simplified manner, excluding criteria such as feasibility and budget impact, which can be incorporated in practice. In the context of HBP revision, such as in Ethiopia,^17^ analysts using this approach initially ranked services based on their cost-effectiveness, and committee members subsequently adjusted the ranking based on other criteria. This approach avoids the implementation challenges of the quantitative approach and provides structure to discussions.

 In our experience and in documented reviews of HBP revision, countries typically use either a qualitative approach or decision-tree approach in their appraisal phase^3,10^ (Table). The difference is subtle and lies in the sequence of criteria discussions, which can influence how evidence is interpreted and recommendations are formed. In the countries we worked in, cost-effectiveness was prominently emphasized in the qualitative approach (such as being listed first, highlighted, or labelled as an “initial priority” on the evidence summary sheet) effectively steering decisions toward this criterion. Consequently, qualitative approaches in practice may lean towards decision-tree logic which begins with an initial ranking of services based on cost-effectiveness.

 An important aspect to consider is cognitive overload. When TWGs are tasked with appraisal of many services, it is questionable whether they can effectively process all evidence on the large number of decision criteria that are typically employed in HBP revisions (eg, eight in Pakistan and ten in both Kyrgyzstan and Rwanda), as shown in the performance matrix. The cognitive overload may imply that TWG members may resort to other ways of sorting their preferences, and key criteria may be overlooked. In contrast, HTA agencies in the United Kingdom^14^ and the Netherlands,^15^ employ a small number of core decision criteria, collect evidence on these, and concentrate the debate on these while providing opportunities to discuss other considerations qualitatively. Future research could explore optimal sets of decision criteria for HBP revision, as perceived by TWGs and AC. Another option to reduce cognitive overload is to use the quantitative approach including scoring and weighting, and we welcome future research to study its use—if implemented adequately—in HBP design.

 Overall, it is difficult to judge the preferred approach to trade-off decision criteria, and analysts should ensure that the chosen method aligns with the intended purpose. For transparency and clarity, analysts are encouraged to explicitly state their selected approach for trading-off decision criteria and reference this classification where applicable.

###  Classifying Services in Priority Classes

 In the countries we worked in, TWGs were instructed to prioritize services into broad priority classes, in the absence of a clear cost-effectiveness threshold or budget constraint (See section 5 for more detail). These priority classes included a high priority class (“services considered essential for the country”), medium priority class (“services that should only be included after all high priorities are included and funding remains available”), and low priority class (“services that should not be implemented”). In various instances, we observed that TWGs have the tendency to classify a large proportion of services as high priority which considerably exceeded the budget constraint – we presented them with (some kind of) a budget constraint for their particular cluster, to make their classification relevant to the AC to develop final recommendations.

## Making Final Recommendations

 In the development of final recommendations to the final decision-maker, the AC integrates the preliminary recommendations from the TWGs in terms of high, medium and low priorities (Table). It may well be the case that the combined set of high priorities exceeds the overall budget constraint, and the AC then may need to make further choices on number of services covered, percentage of population covered and co-payment levels. Note that choices on the population coverage and co-payments levels are of key importance, as they directly impact access to services to disadvantaged populations in terms of eg, area of living or socio-economic status.^6^ The AC may also decide to exempt specific disadvantaged groups from co-payments for all services. Also note that budgets are often uncertain, and analysts can then work with different budget scenarios.

 In this process, the AC may face two challenging situations. Firstly, they may have identified low value services but find it difficult to fully disinvest/exclude these from the HBP because of stakeholders’ interests and opposition. In this situation, they may opt for a “softer” approach that is politically more acceptable, ie, to enhance co-payments levels for these services, or to reduce future coverage levels by choosing to not further invest in these services. Second, the identified high priority services may exceed their budget, and in this situation, they may wish to introduce co-payments, reduce coverage levels, or defer implementation for some services for some years to free up resources to enable funding of more high priority services.^18^ Or alternatively, they may deliberately include too many services and exceed the budget, anticipating that funding will become available, or to put pressure on the decision-maker/Minister of Health or Minister of Finance to make more resources available.

## Use of Cost-Effectiveness Evidence, Thresholds, and Budgets

 In the countries, the assessment and organisation of the appraisal phase by TWGs was much centred around the use of cost-effectiveness evidence. However, this was not applied in a strict formulaic manner, such as in a traditional league table approach which ranks services based on their cost-effectiveness ratio (possibly in conjunction with other criteria) and uses a budget constraint or cost-effectiveness threshold to determine which services should be included in the HBP. The reason is that specific budgets for service clusters are often difficult to define, and very few countries have established a cost-effectiveness threshold for use in HBP revision.^19^ For these reasons, the TWGs did not have clear limits to service inclusion and developed their preliminary recommendations in terms of broad priority classes. The AC then consolidated these recommendations from all TWGs and applied the overall budget constraint (which is usually more clearly defined) to finalize the inclusion of services (Table).

## Discussion

 The appraisal phase in HBP revision is characterized by a complex context of multiple services, multiple criteria, multiple stakeholders, an often-limited evidence base, an uncertain budget constraint and a limited time frame, which takes different forms in different countries. This editorial highlights important areas of cross-country learning on how countries can pragmatically address particular challenges they face. This makes the conduct of a high-quality and legitimate appraisal an art as much as a science.

 The above manifests itself in the different choices countries have made within the model we present. For example, the TWG on multiple sclerosis in Iran identified both in- and exclusions to the HBP which were overall budget-neutral and hence had a clear limit to service inclusion – they could in that sense be specific in their preliminary recommendations to the AC. As another example, the TWG on cancer control in Rwanda initially proposed a broad set of priorities but was requested by the AC to be more specific – when given a budget constraint the TWG then recommended a specific and prioritized set of cancer services. These examples also demonstrate our belief that project teams should present TWGs as much as possible with limits to service inclusion (such as budget constraints or cost-effectiveness thresholds) so they can be specific in the development of their preliminary recommendations.

 The various methodological choices in the appraisal phase (eg, choice of decision-making structure or analyses to trade-off decision criteria) and their potentially large consequences for HBP composition always deserve explicit recognition by those designing HBP revision processes. Their choice may largely be driven by individual preferences and/or experience of (international) analysts, and we argue that these choices should be made based on local needs and the cross-country learning we describe above.

 Our focus on the experiences from Kyrgyzstan, Liberia, Pakistan, Iran, and Rwanda is based on our direct involvement in and detailed observation of HBP appraisal practices in these countries. While we would have liked to provide more examples from other contexts, practices surrounding the appraisal phase are rarely documented in detail in the literature. To build a more comprehensive understanding, we advocate for detailed documentation of appraisal processes in other countries.

 In addition, we encourage research on stakeholder preferences concerning the organization of the appraisal phase, eg, on optimal sets of decision criteria to consider, as well as experimental comparative analyses of appraisal approaches. These insights should be integrated into guidelines for HBP revisions.

## Ethical issues

 Not applicable.

## Conflicts of interest

 Authors declare that they have no conflicts of interest.
